# Investigation on the self-association of an inorganic coordination compound with biological activity (Casiopeína III-ia) in aqueous solution

**DOI:** 10.1186/s13065-016-0213-9

**Published:** 2016-10-21

**Authors:** Alejandro Marín-Medina, Juan Carlos García-Ramos, Lena Ruíz-Azuara, Ernesto Carrillo-Nava

**Affiliations:** 1 Laboratorio de Biofisicoquímica, Departamento de Fisicoquímica, Facultad de Química, Universidad Nacional Autónoma de México, Mexico, D. F. 04510 Mexico; 2 Departamento de Química Inorgánica y Nuclear, Facultad de Química, Universidad Nacional Autónoma de México, Mexico, D. F. 04510 Mexico; 3 Departamento de Fisicoquímica, Instituto de Química, Universidad Nacional Autónoma de México, Mexico, D. F. 04510 Mexico

**Keywords:** Casiopeína, Critical aggregate concentration, Isothermal titration calorimetry, Surface tension, Dynamic light scattering

## Abstract

From studies using different experimental techniques employed to determine the presence of aggregates e.g. isothermal titration calorimetry, surface tension, electrical conductivity, UV–Vis spectrophotometry, dynamic and static light scattering, it is clearly demonstrated that the compound [Cu(4, 4′-dimethyl-2, 2′-bipyridine)(acetylacetonato)H_2_O]NO_3_ (Casiopeína III-ia), promising member of a family of new generation compounds for cancer treatment, is able to auto associate in aqueous media. Physicochemical properties associated with the formation of the aggregates were determined in pure water and in phosphate buffer media in order to simulate physiological conditions. From isothermal titration calorimetry and electrical conductivity measurements we calculated the dissociation constant of the aggregates, *K*_*D*_. For pure water the values obtained in both techniques are 2.73 × 10^−4^ and 5.93 × 10^−4^ M respectively while for the buffer media we obtained 4.61 × 10^−4^ and 1.57 × 10^−3^ M. The enthalpy of dissociation, *∆H*_*D*_, calculated from the calorimetric data shows that the presence of the phosphate ions has an energetic effect on the aggregate stability since in pure water a value of 18.79 kJ mol^−1^ was obtained in comparison with the buffer media where a value 4 times bigger was found (70.48 kJ mol^−1^). With the data collected from these techniques the number of monomers calculated which participate in the formation of the aggregates is around two. From our surface tension, electrical conductivity and UV–Vis spectrophotometry measurements the critical aggregate concentration, *cac*, was determined. For each technique specific concentration ranges were obtained but we can summarize that the *cac* in pure water is between 3 and 3.5 mM and for the buffer media is between 3.5 and 4 mM. Dynamic light scattering measurements provide us with the hydrodynamic diameter of the aggregates and from static light scattering measurements we determined the molecular weight of the Casiopeína III-ia aggregates to be of 1000.015 g mol^−1^ which is two times the molecular weight of the Casiopeína III-ia molecule. This value is in agreement with the number of monomers which participate in the formation of the aggregates obtained from isothermal titration calorimetry and electrical conductivity data analysis.

## Background

**Fig. 1:**
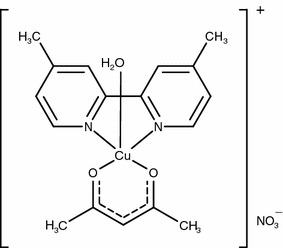
Chemical structure of [Cu(4, 4′- dimethyl-2, 2′-bipyridine)(acetylacetonato)H_2_O]NO_3_ (Casiopeína III-ia)

The coordination complex [Cu(4, 4′-dimethyl-2, 2′-bipyridine)(acetylacetonato)H_2_O]NO_3_, Casiopeína III-ia, (Fig. 1) is a member of a group of compounds patented and registered under the generic name of Casiopeínas [1, 2]. They are metal complexes which have been developed as a new generation of copper coordination complexes to be used as pharmaceuticals, diagnostic agents or as chemotherapeutic drugs. Some members of the family of Casiopeínas have shown cytotoxic, genotoxic and antineoplastic activity both in in vitro and in vivo studies [3–5]. Casiopeína III-ia is one of the most promising members of this family of compounds and it is currently under phase I clinical trials. Although the mechanism of action is not known with great detail at the molecular level, several experimental results seem to indicate that the two main factors for the cytotoxic induction on human tumor cell are: (i) the generation of reactive oxygen species and (ii) direct interaction with DNA. Experimental observations showed that different members of the family of the Casiopeínas present nuclease activity in contact with DNA, comparable to that shown by other metal complexes [6]. These results allowed to design and perform an experiment of the whole genetic expression of tumor cells exposed to Casiopeínas, where it was found that particular pathways related with the cell cycle, gene expression, cellular growth, proliferation and cell death were affected, all of them related with oxidative stress and DNA damage [7].

The aim of studies related with the formation of specific interactions between the Casiopeínas with important biological targets such as DNA or proteins is to further advance in the understanding of the possible mechanism of action of these compounds, which will provide us with information in order to guide an intelligent development of drugs with an increased specificity and reduction of undesirable side effects. During the course of studies involving the formation of protein—Casiopeína III-ia complexes we have found that at the concentrations in which our experiments were being carried out Casiopeína III-ia was self-associating in the buffer media employed to simulate physiological conditions, a feature which had not being identified to our knowledge. Since it is known that the surface and self-association properties of pharmacologically active compounds play an important part in the mechanisms of the biological activity of such compounds [8], it is of utmost importance to study and characterize the different physicochemical properties of the Casiopeína III-ia aggregates. It is therefore of interest to determine the critical aggregation concentration, *cac*, the equilibrium constant of dissociation, *K*_*D*_, the enthalpy of dissociation, *∆H*_*D*_, and the aggregation number of the Casiopeína III-ia aggregates using different experimental techniques.

The findings we report in this work are relevant for studies involving the formation of protein—Casiopeína III-ia complexes. It is known that protein—substrate interactions are governed by the specificity and selectivity of the binding site of the protein to the substrate [9]. Experimental conditions must then be precisely established in order to carry out studies where the precise state of aggregation of the substrate is known so the physicochemical properties which characterize the protein—Casiopeína III-ia complex (formation constant, formation enthalpy, stoichiometry, etc.) are unambiguously defined for the species involved in the formation of the complex. These studies are in need in order to elucidate the Casiopeína III-ia mechanism of action or to use proteins as Casiopeína III-ia carriers.

## Experimental section

### Materials and solutions

Casiopeína III-ia was synthesized following the procedure reported in the literature and was obtained with a purity higher than 99 % [1, 2]. Milli-Q water with a resistivity of 18.1 MΩ cm^−1^ was used to prepare all solutions, the salts used to prepare the two buffer solutions studied in this work were Na_2_HPO_4_∙7H_2_O, NaH_2_PO_4_∙H_2_O and KNO_3_, all analytical grade and purchased from Baker. The range of the concentrations studied was limited from 0.5 up to 10 mM due to the poor solubility that Casiopeína III-ia presents in water and in the two buffer media reported in this work.

From previous studies we have found that the phosphate ion can replace one or both of the organic ligands which make up Casiopeína III-ia. Following the change in the absorbance spectrum by UV–Visible spectrophotometry we have determined that the replacement process is not instantaneous but it takes around three weeks. In order to ensure that the physicochemical properties of Casiopeína III-ia are properly determined in all our measurements, freshly prepared solutions of Casiopeína III-ia were employed during the course of our studies.

### Isothermal Titration Calorimetry (ITC)

Heats of dissociation of the Casiopeína III-ia aggregates in pure water and in phosphate buffer 0.1 M, pH 7.4 were measured at 25 °C using a VP ITC instrument (Microcal, Northampton USA). The syringe of the calorimeter was filled with 10 mM Casiopeína III-ia solutions of either one of the two aqueous media mentioned above and titrated into the cell which only contains the matching media in order to avoid additional heat development due to aqueous media mismatch and the corresponding heat of dilution. The Casiopeína III-ia and the different aqueous media solutions were degassed before being loaded into the syringe and the reaction cell of the calorimeter. Titrations of 5 µL spaced by 700 s were carried out. The calorimetric signals were integrated to obtain the corresponding heats associated with each addition of the Casiopeína solution into the matching aqueous media with the Origin 7.0 (OriginLab Corporation, Northampton, U. S. A.) software macros supplied by the manufacturer.

### Surface tension measurements

The concentration dependence of the surface tension of the aqueous solution and the phosphate 0.1 M, KNO_3_ 0.1 M and pH 7.4 solution of Casiopeína III-ia were determined by means of a K12 Krüss tensiometer (Hamburg, Germany) which employs the Du Noüy ring method. All the measurements were carried out at a constant temperature of 25 ± 0.1 °C and collected from three independent solution preparations.

### Conductivity measurements

The electrical conductivity of the aqueous solutions and the phosphate 0.1 M, KNO_3_ 0.1 M and pH 7.4 solutions of Casiopeína III-ia was determined using an Oakton CON 110 conductometer (Oakton Instruments, Vernon Hills, U. S. A.) at 25 ± 0.1 °C which has an accuracy of ±1 % in the full scale and the alternating current supplied to the bridge has a frequency of 2 kHz. During the determination of the conductivity of the different Casiopeína III-ia solutions the samples were stirred and between measurements of the different solutions the conductivity cell was cleaned thoroughly with Milli-Q water and rinsed with a small amount of the sample of the new solution from which conductivity data was to be measured.

From plots of the observed molar conductivity, Λ_obs_, as a function of the square root of the concentration and establishing an equilibrium constant for the formation of aggregates from monomer species, it is possible to fit the experimental data to the equations relating the molar conductivities of each of the species involved in the equilibria as well as the equilibrium constant, and the aggregation number. We have performed the data analysis of the observed molar conductivity following the procedure described in detail by Streng et al. [10].

### UV–Visible spectrophotometry

The absorbance of the colored Casiopeína III-ia solutions was measured using a Cary 50 Bio spectrophotometer (Varian, Australia) at 25 ± 0.1 °C. The concentration dependence of the absorbance for the solutions in the different media was collected from three different solution preparations. Between measurements of the different solutions the optical cell was cleaned thoroughly with Milli-Q water and rinsed with a small amount of the sample of the new solution from which absorbance data was to be collected.

### Dynamic light scattering (DLS) and Static light scattering (SLS) analysis

DLS measurements were performed employing a Zetasizer µV (Malvern, Worcestershire, United Kingdom) light scattering instrument which is equipped with a 60 mW He–Ne laser, operating at a wavelength of 830 nm. Light intensity was collected at an angle of 90° and at a fixed temperature of 25 ± 0.1 °C in a quartz cuvette. Size distribution was obtained by multiple data acquisitions (15) of 40 s each with a total of five replicates. The concentration of Casiopeína III-ia in the solutions used for the size determination of the aggregates was 10 mM. Data analysis was carried out using the Zetasizer v 7.11 software.

In order to determine the molecular weight of the Casiopeína III-ia aggregates, SLS measurements were performed with the same equipment as in the case of the DLS experiments described in detail above. For each concentration the data acquisition was collected from 10 measurements with a duration of 30 s each, with a total of five replicates. Data analysis was also carried out with the Zetasizer v 7.11 software supplied with the instrument.

## Results and discussion

### Isothermal titration calorimetry

**Fig. 2:**
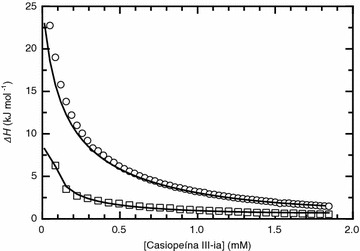
Heat changes as a function of the total Casiopeína III-ia concentration in the calorimetric cell from ITC measurements carried out in two aqueous media. *Circles* correspond to the solute dissolved in the phosphate 0.1 M and pH 7.4 media and titrated into the same buffer while *squares* represent the solute dissolved in pure water and titrated into the same solvent. The* lines* correspond to the calculated heat changes using optimized values of *K*_*D*_, *∆H*_*D*_ and the stoichiometry of the aggregate formation equilibria (*χ* ^*2*^ = 0.0513 for Casiopeína dissolved in the phosphate media and *χ* ^*2*^ = 0.086 for Casiopeína dissolved in pure water)

Through isothermal titration calorimetry the energetics associated with the dissociation of Casiopeína III-ia aggregates in pure water and the phosphate 0.1 M, pH 7.4 media were determined. Figure 2 shows the resulting thermogram for both aqueous media where it is seen that the dissociation process is endothermic. As the concentration of Casiopeína III-ia progressively increases in the calorimetric cell during the course of the experiment, the resulting heat from the dissociation process decreases tending to a value of zero. As it is observed, the dissociation process of the Casiopeína III-ia aggregates follows the same trend in both aqueous media but the dissociation energy is remarkably different for each case. The amount of heat evolved after each addition of the solute in the phosphate 0.1 M, pH 7.4 media is around 4 times higher in comparison to that obtained when titrating into pure water. In both cases the shape of the curve indicates that the number of monomers which participate in the formation of the aggregates is low, since aggregates with high number of monomers display normally a high cooperativity during the dissociation process and also the curve follows a sigmoidal trend which is centered near the critical aggregation concentration [11].

In order to obtain the thermodynamic parameters of the dissociation process the data was analyzed via an iterative nonlinear least square algorithm using a dissociation model where the fitting parameters are *K*_*D*_, *∆H*_*D*_ and the aggregation number [11, 12]. Figure 2 also shows the curves obtained using the values of the parameters from the best fit to the dissociation model, which show a good description of the theoretical model to the experimental data. The resulting values for *K*_*D*_, *∆H*_*D*_ and the aggregation number are 2.73 × 10^−4^ ± 0.3 × 10^−4^ M, 18.79 ± 1.21 kJ mol^−1^ and 2.2 respectively for Casiopeína dissolved in pure water and 4.61 × 10^−4^ ± 0.6 × 10^−4^ M, 70.48 ± 0.51 kJ mol^−1^ and 2.0 for Casiopeína dissolved in the buffer media. The number of monomers participating in the formation of the aggregates is very low as one would expect from the shape of the titration curve, as described previously. Data analysis shows that the aggregates are dimers which possess low dissociation constants in both aqueous media. The main difference between the two systems studied is the enthalpy of dissociation of the aggregates in the different aqueous media. The only fact responsible for such difference in enthalpy is the presence of the phosphate ions and therefore it must contribute energetically to the stabilization of the dimers, one could even speculate that the phosphates could participate directly in the aggregation process. This hypothesis is based in the fact that from our dynamic light scattering studies we have found that the hydrodynamic radii of the aggregates in the phosphate media are bigger in comparison to the ones present in pure water and is further elaborated in the article in another section (see discussion in ‘‘Dynamic light scattering and static light scattering analysis’’ section).

### Surface tension measurements

**Fig. 3:**
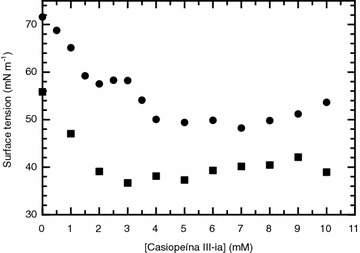
Surface tension versus solute concentration for the two Casiopeína III-ia aqueous media reported in this work. *Circles* correspond to the solute dissolved in pure water while the *squares* represent the solute dissolved in the phosphate 0.1 M, KNO_3_ 0.1 M and pH 7.4 media

For the two different aqueous media studied it was found that Casiopeína III-ia exhibits surface activity, since it is able to modify the surface tension as shown in the plots of surface tension as a function of concentration of the solute in Fig. 3. Its surface activity is not so strong as the one displayed by typical surfactant molecules which are able to decrease the value of surface tension by 30 mN m^−1^or more nevertheless, it is able to decrease the surface tension of pure water and the buffer solution media by 23 and 19 mN m^−1^ respectively. The concentration dependence of the surface tension for the two systems follows a different trend: (i) for pure water the surface tension decreases dramatically in the concentration range 0 to 1.5 mM followed by a small region between 1.5 and 3 mM where the surface tension remains constant, decreasing once more and remaining constant from around 4 mM up to the final concentration and (ii) in the case of the buffer solution the surface tension decreases dramatically in the concentration range 0 to 3 mM, from where the surface tension remains constant (see Fig. 3).

The lack of a concentration dependence of the surface tension after a certain solute concentration is reached is associated with the ability of the solute to form aggregates in the bulk of the solution, for surfactants which are able to self-associate into micelles this concentration is called critical micelle concentration (*cmc*). In the case that the aggregates are not micelles the term critical aggregation concentration (*cac*) is more appropriate. We have employed a Gibbs adsorption isotherm analysis in order to determine the *cac* value in each aqueous media. As shown in Fig. 3, Casiopeína III-ia has different *cac* values for each of the aqueous media studied in this work. For pure water the *cac* value is 4.09 mM while for the phosphate 0.1 M, KNO_3_ 0.1 M and pH 7.4 media it is 2.87 mM. As seen from the chemical structure of Casiopeína III-ia in Fig. 1, the compound has two organic ligands which are coordinated with the metallic center. From these two ligands 4, 4′-dimethyl-2, 2′-bipyridine has a low solubility in aqueous media which makes it the hydrophobic element of the coordination compound while the acetylacetonato moiety has a higher solubility, following these line of thought Casiopeína III-ia has the right chemical moieties to present surface activity. Regarding the differences in the *cac* values in each aqueous media the presence of the phosphate ions and the increment in the ionic strength promotes the aggregation process since the *cac* value is roughly half the value obtained in the pure water media. As it will be discussed in other sections of this article, the phosphate ions have a big influence in the physical characteristics of the Casiopeína III-ia aggregates.

### Conductivity measurements

**Fig. 4:**
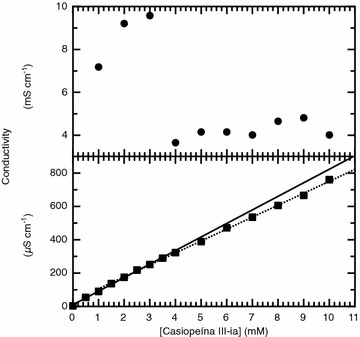
Electrical conductivity dependence with concentration of the solute. The *upper panel* corresponds to Casiopeína III-ia dissolved in the phosphate 0.1 M, KNO_3_ 0.1 M and pH 7.4 media. The *lower panel* represents data collected for Casiopeína III-ia dissolved in pure water. Two linear concentration dependencies can be seen (R^2^ = 0.998 for the first region and R^2^ = 0.996 for the second region), each corresponds to the different electrical conductivity concentration dependence that the free and the aggregate species possess

The concentration dependence profile of the electrical conductivity of the aqueous and the buffer Casiopeína III-ia solutions is shown in Fig. 4. In the case of the pure water solutions (Fig. 4, lower panel) the conductivity increases monotonically with the concentration of the solute which is the common trend observed for electrolyte solutions. Careful analysis of the full curve shows that there are two different linear trends which describe the observed data in the complete concentration range of our study. At low concentrations it is the concentration dependence of Casiopeína III-ia monomers which is seen. At a concentration range between 3 and 3.5 mM a shift is observed in the linear electrical conductivity dependency with concentration. This behavior is associated with the formation of Casiopeína III-ia aggregates since no chemical reaction can occur in the conditions in which our study has been carried out. The difference in the electrical conductivity dependency between the monomers and the aggregates is due to the fact that the mobility and the charge of these species are not the same [10].

In the case of the phosphate 0.1 M, KNO_3_ 0.1 M and pH 7.4 media, the observed trend is dramatically different (Fig. 4, upper panel). The conductivity values are 1000 higher than those observed for Casiopeína III-ia dissolved in pure water which is the result of the presence of KNO_3_, which is a strong electrolyte, in the media. At low concentrations the normal monotonical increase of electrical conductivity with concentration is observed, but between the concentration range of 3 and 4 mM the electrical conductivity falls sharply and then remains constant. The observed phenomenon is a mere result of the dramatic change in the charge and the mobility of the aggregates in comparison to the free Casiopeína III-ia molecules. The concentration range where the property changes significantly corresponds to the critical aggregation concentration.

As it was mentioned in the “Conductivity measurements” section, the data analysis of the observed molar conductivity was done following the procedure described by Streng et al. [10]. The procedure described briefly is the following: data analysis is made considering that the monomer and the aggregate are strong electrolytes therefore the molar conductivity of both species can be expressed as linear functions of the square root of the concentration of each of the species, described by the following equation

1 $$\begin{aligned} \Lambda _{{{\text{obs}}}} = & \frac{{\left( {c_{T} - nc_{M} } \right)}}{{c_{T} }}\left( {a - b\sqrt {c_{T} - nc_{M} } } \right) \\ & \quad + \frac{{c_{M} }}{{c_{T} }}\left( {a^\prime - b^\prime \sqrt {c_{M} } } \right), \end{aligned}$$

where *c*_*T*_ is the total concentration of the sample, *c*_*M*_ is the concentration of the aggregate, *a* is the limiting molar conductivity of the monomer i.e. the molar conductivity at infinite dilution, *b* is the Kohlrausch constant for the monomer, *a′* is the limiting molar conductivity of the aggregate and *b′* is the Kohlrausch constant for the aggregate. The limiting molar conductivity of the monomer and the Kohlrausch constant of the monomer are obtained from a linear fit of the conductivity data in the low concentration range, below the critical aggregation concentration. The remaining parameters in Eq. 1 are obtained through a non-linear least squares regression fit of the observed molar conductivity data dependency with the square root of the concentration.

**Fig. 5:**
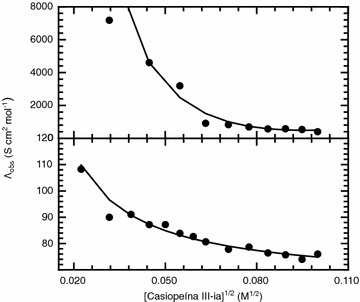
Molar conductivity concentration dependence for Casiopeína III-ia dissolved in two different aqueous media. The *upper panel* corresponds to the solute dissolved in the phosphate 0.1 M, KNO_3_ 0.1 M and pH 7.4 media while the *lower panel* corresponds to the solute dissolved in pure water. The* lines* correspond to the best fit achieved with parameters summarized in Table 1

For both systems the observed molar conductivity, Λ_obs_, as a function of the square root of the concentration is shown in Fig. 5. Strong electrolytes which do not form aggregates in aqueous media show a linear dependency. We found that Casiopeína III-ia is a potential electrolyte and the departure from linearity suggest that aggregation has occurred in the concentration range of study. Fitting the observed molar conductivity we have calculated the dissociation constant of the aggregates as well as the aggregation number. As seen from Fig. 5 there is a good agreement between the observed experimental data and the theoretical description. In Table 1 the parameters obtained from fitting the observed molar conductivity are summarized. The aggregation number, *n*, for both aqueous media obtained from this analysis are in good agreement with the ones we found from our dissociation studies using the ITC technique, indicating that the aggregates are formed with a low number of monomers. Also in good agreement with our ITC studies are the *K*_*D*_ values obtained for both aqueous media.

**Table 1:** Fitted values of the parameters of the molar conductivity dependency with concentration equation of Casiopeína III-ia in different aqueous media

Parameter	Aqueous media	
	Pure water	Phosphate 0.1 M, KNO_3_ 0.1 M and pH 7.4
*n*	2	2
*K*_*D*_	5.93 × 10^−4^ M	1.57 × 10^−3^ M
*a*	102.6	1020
*b*	38.6	14,961
*a′*	0.0017	0.069
*b′*	0.109	0.870
*χ* ^*2*^	0.0016	0.0481

*n* aggregate number; *K*_*D*_ dissociation constant; *a* limiting molar conductivity of monomer; *b* constant for monomer; *a′* limiting molar conductivity of aggregate; *b′* constant for the aggregate; *χ* ^*2*^ is the value of the minimization function (Chi squared test) employed to find the best parameter set which describes the experimental data

### UV–Visible spectrophotometry

**Fig. 6:**
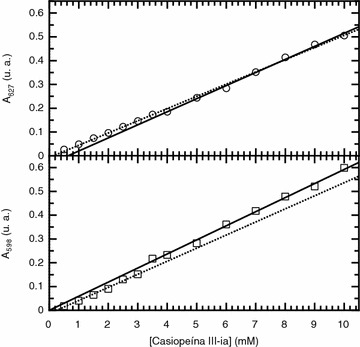
UV-Vis absorbance dependence with concentration of the solute. The *upper panel* corresponds to Casiopeína III-ia dissolved in the phosphate 0.1 M, KNO_3_ 0.1 M and pH 7.4 media while the *lower panel* represents data collected for Casiopeína III-ia dissolved in pure water. Both systems follow an increment of absorbance with solute concentration but two linear dependencies are required for the full solute concentration range (R^2^ = 0.999 for the lower concentration range and R^2^ = 0.998 for the upper concentration range of Casiopeína dissolved in phosphate media. R^2^ = 0.996 for the lower concentration range and R^2^ = 0.998 for the upper concentration range of Casiopeína dissolved in pure water)

For the aqueous and the phosphate 0.1 M, KNO_3_ 0.1 M and pH 7.4 media the UV–Visible absorbance spectrum was obtained in order to determine the best wavelength to follow the concentration dependency of the absorbance. For pure water it showed an absorption maxima centered at 598 nm while for the buffer media it is shifted to 627 nm. The absorbance at these wavelengths was then followed for each system as a function of concentration. Typical monotonic absorbance dependency with concentration was observed but as in the case of electrical conductivity different lineal behaviors describe the whole concentration range as shown in Fig. 6. The observed shift in absorbance must be due to effects on the effective dielectric constant of the aggregates which modify the excited states of the molecules and not to the presence of turbidity, which was not observed. In fact, the samples were kept at room temperature for a month and there was no indication of precipitation. The concentration where there is a shift in the absorbance dependency with concentration is then considered as the critical aggregation concentration. For the pure aqueous media this corresponds to a concentration range between 3 and 3.5 mM and 3.5 and 4 mM for the phosphate 0.1 M, KNO_3_ 0.1 M and pH 7.4 media.

### Dynamic light scattering and static light scattering analysis

**Fig. 7:**
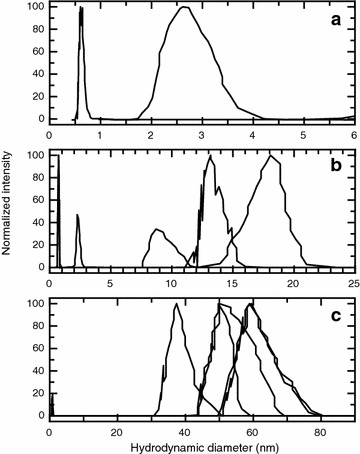
Dynamic light scattering analysis of Casiopeína III-ia monomers and aggregates in different aqueous media, **a** pure water, **b** phosphate 0.1 M and pH 7.4 and **c** phosphate 0.1 M, KNO_3_ 0.1 M and pH 7.4

Our dynamic light scattering studies reveal that Casiopeína III-ia forms aggregates and the size of the observed aggregates is different in each of the aqueous media studied in this work. The DLS studies were performed in systems where Casiopeína III-ia is dissolved in: (i) pure water, (ii) phosphate 0.1 M, and pH 7.4 and (iii) phosphate 0.1 M, KNO_3_ 0.1 M and pH 7.4 media. For the different aqueous media studied it is found that the populations of the aggregates are not homogeneous, but within the polydispersity of the media there are well differentiated populations which we have assigned in the following way (Fig. 7): (i) for all the aqueous media there is a population with a mean hydrodynamic diameter around 0.66–0.95 nm which corresponds to the monomers i.e. singly dispersed Casiopeína III-ia molecules. This value corresponds well to the molecular diameter of 0.808 nm estimated from van der Waals radii calculated with the help of the software Marvin v. 15.8.31, 2015 which was used together with its calculator plugins (ChemAxon, http://www.chemaxon.com) and the molecular diameter of 0.82 nm reported in the literature and obtained from an X-Ray diffraction characterization [13], (ii) there are small aggregates present in the systems where Casiopeína III-ia is dissolved in pure water and in the phosphate 0.1 M and pH 7.4 media, and which have a hydrodynamic diameter centered around 2.70 nm. For the phosphate 0.1 M, KNO_3_ 0.1 M and pH 7.4 media interestingly this population is not present and would indicate that the ionic strength of the media plays and important role in the aggregate size and (iii) there are bigger aggregates with hydrodynamic diameters centered around 9.15, 13.17, 18.45, 39.89, 58.87 and 67.03 nm which are present only in the systems where Casiopeína III-ia is dissolved in the buffered aqueous media. Interestingly the bigger aggregates are present when KNO_3_ 0.1 M is added in the media. These facts clearly indicate that the ionic strength of the media has a strong influence over the solvation sphere around the Casiopeína III-ia aggregates.

For the buffered media these observations seem to be in contradiction to the results and analysis we obtained in calorimetry and conductometry regarding the size of the aggregates since the DLS results indicate that the aggregate size increases with ionic strength and therefore one would expect a higher aggregation number for these systems, while the other results indicate that the aggregates have a low aggregation number. Our hypothesis in order to reconcile these conflicting observations is the following: Through our calorimetry and conductometry analysis we have determined the number of Casiopeína III-ia molecules which participate in the formation of an aggregate, and this number is around two. In the presence of phosphate and KNO_3_ our DLS results indicate that the Casiopeína III-ia dimer is surrounded by phosphate ions which increase the hydration layer around the aggregate due to the fact that they are much bulkier than the water molecules. Either a coordination between the metallic center of the Casiopeína III-ia and the phosphate and/or through electrostatic interactions this hydration layer is bigger in comparison to the one formed when the solute is dissolved in pure water. It is known that the copper (II) ion dissolved in phosphate buffer at a pH of 7.4 is not in the form of the aquacation or hydrolytic species but as the [Cu(HPO_4_)] specie [14]. In the Casiopeína III-ia coordination compound the copper (II) ion is able to coordinate with the phosphate species (HPO_4_^2−^ or H_2_PO_4_^−^) replacing the water molecule and therefore it is able to form hydrogen phosphate bridges with other Casiopeína III-ia aggregates. These hydrogen phosphate bridges have been reported for coordination compounds with copper (II) as the metallic center [15–17], and also with other coordination compounds where the metallic centers are vanadium and zirconium [18, 19]. These hydrogen phosphate bridges could also explain the reason why in our calorimetric studies we found a higher value of *∆H*_*D*_ for the dissociation of the Casiopeína III-ia aggregates in the buffer media in comparison with the pure water media.

Adding an electrolyte (KNO_3_) to the phosphate media promotes the growth of the solvation layer due to a shielding effect. It would seem that more layers of phosphate are able to build up around the Casiopeína III-ia dimer or/and that neighboring aggregates with their solvation sphere are able to group through electrostatic interactions or hydrogen bonding. It is reported in the literature that some copper (II) coordination compounds are able to form double anti parallel polymer chains which are kept together by strong phosphate-water bonds [14, 20]. Although from our findings it is clear that Casiopeína III-ia does not form such polymers it is undoubted that the phosphate media is responsible for the increase in size of the observed aggregates. The fact that the properties of the aggregate are different or are altered by the addition of ions has been well reported in the literature [21, 22].

**Fig. 8:**
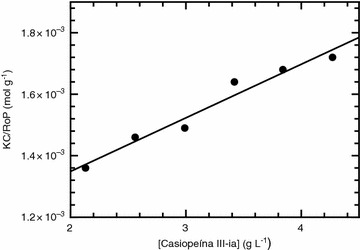
Debye plot of Casiopeína III-ia dissolved in pure water. *Circles* represent the experimental data from the Static Light Scattering measurements while the *line* corresponds to the fitted curve obtained from fitting the experimental data to Rayleigh’s equation (R^2^ = 0.978)

From the SLS measurements it is possible to determine the molecular weight of the aggregates in solution. Polydispersity of a sample produces bad estimates for the determination of the molecular weight. Since we found a much lower polydispersity in the case where Casiopeína III-ia is dissolved in water we centered our efforts into characterizing the molecular weight of the resulting aggregates only for this system. The molecular weight of the aggregates is estimated by measuring the scattered light of different concentrations of the sample and applying Rayleigh’s equation, which describes the intensity of the scattered light from a particle in solution [23].

2 $$\frac{KC}{{R_{\varTheta } }} = \left( {\frac{1}{M}} \right) + 2A_{2} C$$

where *K* is the optical constant, *C* is the concentration of the sample, *M* is the sample molecular weight, *R*_*Θ*_ is the Rayleigh ratio i.e. the ratio of the scattered light to incident light of the sample and *A*_*2*_ is the second virial coefficient. Since we are interested in finding the molecular weight of the aggregates the concentration range of our studies involves concentrations higher than the critical aggregation concentration (from 5 to 10 mM). The results obtained from our SLS studies are depicted in Fig. 8 and as it is seen, the intensity of the scattered light from the samples is proportional to the concentration of the Casiopeína III-ia. From Rayleigh’s equation the molecular weight of the Casiopeína aggregates can be calculated from the intercept at zero concentration. The resulting molecular weight of the aggregates was determined to be 1000.015 ± 51 g mol^−1^, and given that the molecular weight of the Casiopeína III-ia molecule is 444.92 g mol^−1^ it results then that the number of monomers participating in the aggregate formation is 2.24. This numerical value is in agreement with our other determinations of the number of aggregation employing isothermal titration calorimetry and data analysis of the molar conductivity dependency with concentration of the solute.

Since Casiopeína III-ia is a member of around 100 compounds in the family of the Casiopeínas our results indicate that several members of this family could be capable of forming aggregates at a certain concentration due to the nature of the ligands employed to synthesize these copper coordination compounds. The need to carry out physicochemical studies in order to determine the aggregation properties of this compounds is of great importance since other compounds of the family of the Casiopeínas have also shown antitumor and anti-protozoan activity [7, 24–26].

## Conclusions

Based in the data collected from the different experimental techniques employed in this study it is concluded that Casiopeína III-ia is able to self-associate in aqueous media at 25 °C. The physicochemical parameters associated with the formation of these aggregates: critical aggregation concentration, aggregation number, dissociation constant and the enthalpy of dissociation were determined. There is a very good agreement for the values of *K*_*D*_ and the aggregation number obtained from data analysis carried out from our electrical conductivity measurements and isothermal titration calorimetry. From our studies we determined that for pure water the number of monomers participating in the aggregates is low (around two). This result is in agreement with reported aggregation numbers of several non-peptide surface active drugs in water, whose aggregation numbers vary from 3 to 12 [27, 28].

The presence of the phosphate and electrolytes does not change the number of Casiopeína III-ia molecules which aggregate but they have an important effect in energetic terms and in the formation of a bigger hydration shell around the aggregates. With the information we have collected and analyzed from our studies using different experimental techniques we are not able to establish how the monomers are interacting to form the aggregates but due to the electrolyte nature of Casiopeína III-ia one can assume that the likely interactions should include cation–cation dimer, hydrogen bonding or π−π interactions. In fact, it is known from X-ray diffraction that many members of the Casiopeína family form in the solid state stacked structures stabilized through π–π interactions between the bipyridine or the phenanthroline moieties of the molecule [29, 30].

## Abbreviations

Casiopeína III-ia: [Cu(4, 4′-dimethyl-2, 2′-bipyridine)(acetylacetonato)H_2_O]NO_3_
SLS: static light scattering
DLS: dynamic light scattering
cac: critical aggregation concentration
ITC: isothermal titration calorimetry
*K*_*D*_: dissociation constant
*∆H*_*D*_: enthalpy of dissociation
Λ_obs_: observed molar conductivity
a: limiting molar conductivity of the monomer
b: Kohlrausch constant for the monomer
a′: limiting molar conductivity of the aggregate
b′: Kohlrausch constant for the aggregate
